# Ewe Wastage in New Zealand Commercial Flocks: Extent, Timing, Association with Hogget Reproductive Outcomes and BCS

**DOI:** 10.3390/ani11030779

**Published:** 2021-03-11

**Authors:** Kate J. Flay, Anne L. Ridler, Chris W. R. Compton, Paul R. Kenyon

**Affiliations:** 1 Department of Veterinary Clinical Sciences, City University of Hong Kong, Kowloon, Hong Kong, China; 2 School of Veterinary Sciences, Massey University, Palmerston North 4410, New Zealand; A.L.Ridler@massey.ac.nz (A.L.R.); C.W.Compton@massey.ac.nz (C.W.R.C.); 3 School of Agriculture and Environment, Massey University, Palmerston North 4410, New Zealand; P.R.Kenyon@massey.ac.nz

**Keywords:** ewe, hogget, wastage, longevity, mortality, premature culling, reproduction, body condition score (BCS)

## Abstract

**Simple Summary:**

Ewe wastage is the combination of on-farm mortality and premature culling. Increased wastage results in a reduction in flock productivity and profitability, yet internationally, there is limited research on actual wastage incidence and causes in commercial flocks. This study reports both lifetime wastage and detailed annual wastage for 13,142 ewes from four cohorts on three commercial New Zealand farms. This study also describes the relationship between ewe pre-mating body condition score and wastage. Of the 13,142 enrolled ewes, 50.4% and 40.0% exited their respective flocks due to premature culling and on-farm dead/missing, respectively. Annual mortality incidence ranged from 3.5 to 40.2%. Wastage as a hogget was identified as an area in which improvements can be made to reduce overall wastage. Pre-mating body condition score was a predictor of wastage, with ewes with greater pre-mating body condition score having lower odds of wastage due to both premature culling and mortality. Therefore, farmers should focus on improving the body condition score of their ewes prior to breeding.

**Abstract:**

Ewe wastage is the combination of on-farm mortality and premature culling. Internationally, there is limited research on actual wastage incidence and causes in commercial sheep flocks. To the authors’ knowledge, this is the first study that reports both lifetime wastage and detailed annual wastage in a sample of commercial New Zealand flocks. This study utilized data collected from 13,142 ewes from four cohorts on three commercial New Zealand farms (Farm A 2010-born, Farm A 2011-born, Farm B, Farm C), during the period 2011–2017, as they aged from replacement hoggets to 6-year-old ewes (Farm A and Farm B) or 3-year-old ewes (Farm C). Data collection visits occurred at three or four key management times each year, namely pre-mating, pregnancy diagnosis, pre-lambing and weaning. At each visit, body condition score (BCS) was assessed and any ewes that were culled or had died on farm were recorded. As this was a lifetime study, each ewe was assigned an outcome and corresponding ‘exit age’. By the end of the study, all ewes that had exited their respective flocks, were classified as either prematurely culled, or dead/missing, or if still in the flock, as censored, and either the exact date or interval in which they exited the flock was recorded. Semi-parametric competing risk (premature culling vs. dead/missing), interval-censored survival models were developed to: 1. describe the association between hogget reproductive outcomes and risk of subsequent wastage, and 2. assess pre-mating BCS as a predictor of wastage in that production year. Of the 13,142 enrolled ewes, 50.4% exited their respective flocks due to premature culling and 40.0% due to on-farm dead/missing, giving a total of 90.4% that exited due to wastage. Annual mortality incidence ranged from 3.5 to 40.2%. As a hogget, wastage incidence ranged from 7.6 to 45.4%. Pregnancy or rearing a lamb as a hogget did not increase risk of subsequent wastage. In all years, pre-mating BCS was a predictor of ewe wastage, with odds of wastage lower with increasing BCS. Therefore, farmers should focus on improving pre-mating BCS to 3.5/5.0 by assessing ewe BCS at weaning, allowing poorer-BCS ewes to be managed to gain BCS before re-breeding.

## 1. Introduction

Productive longevity is the ability of a ewe to survive and be productive until she is culled for age. In New Zealand commercial flocks, the age at which ewes are culled for age varies between farms, but is typically approximately six to seven years of age [1], similar to flocks internationally [2,3]. Ewe wastage is defined as the combination of both on-farm mortality and premature culling [4]. Premature culling is where a ewe is culled prior to the potential end of her productive lifespan.

Increased ewe wastage results in a reduction in farm productivity and subsequent ability to generate profit [1,4]. However, there is limited research on actual wastage rates and causes in commercial ewe flocks in New Zealand [1] and internationally [2,5,6]. To maintain consistent breeding ewe flock numbers, replacement ewe lambs (hoggets) are required, with numbers of replacements needed equaling the combined annual total of ewes that are culled for age and ewes that are lost due to wastage. Therefore, flocks that have higher ewe wastage rates require an increased proportion of replacement hoggets. Rearing additional replacement hoggets to a productive age (or purchasing additional replacements) incurs a number of costs [1,2,7], including fewer sale lambs, increased management and feed costs, reduced selection pressure, potential biosecurity risks and increased greenhouse gas emissions. In addition, the reproductive performance of ewes increases as they age [8], so having an unnecessarily high proportion of younger ewes in a flock, due to increased wastage, reduces overall flock productivity due to a lower average flock age [1]. Using a bioeconomic model, Farrell reported that for a New Zealand North Island Hill Country sheep farm with 21% of the flock lost annually due to wastage, a reduction in wastage to 5% could increase cash profit by 33% [1]. Combined, this indicates that it is important for replacement hoggets to remain in a flock, and be productive, for a sufficient period to be economically efficient [6,9,10].

It is important to note variability and potential inaccuracy in recording wastage rates in extensively managed commercial flocks [2,5]. This is likely due to farm topography and flock size, farm management structure, degree of human contact with individual ewes within the flock, and perceived likelihood of wastage [11,12,13]. In addition, in extensive flocks, ewes are typically managed as a flock, with management decisions based on overall flock needs rather than individual animal needs per se. This presents a particular challenge when investigating wastage as ewe numbers are typically based on flock totals at key times of the year (for example shearing) and, in some instances an annual stock reconciliation, rather than individual ewe data. However, the introduction of electronic identification (EID) tags for use in sheep has provided farmers with a relatively straightforward means of tracking individual ewes within a flock, if they choose to utilize it [3]. To date, rates of use of this technology in New Zealand are relatively low, 23.5% of farmers in 2014 [14], contrasting with some overseas sheep producing countries such as in those within the European Union and the United Kingdom where use is mandatory. This technology could be used to more accurately monitor individual ewe wastage.

In New Zealand, reported annual ewe mortality rates range from 2.8 to 27.0% [15,16,17,18,19], with an increased incidence of ewe mortality reported over the lambing period [17]. Comparable rates have been reported in countries such as Australia [5,7,20], the United Kingdom [21,22] and Ireland [23]. Ewe mortality has additional costs when compared to premature culling, as cull sale-value is not obtained and there are likely increased welfare costs associated with on-farm mortality [24]. To the authors’ knowledge, there are no published reports directly examining the premature culling policies in New Zealand commercial flocks. However, it is likely that ewes are culled for similar reasons as reported internationally, namely known poor performance (for example non-pregnant), predicted poor performance (for example ewes whose lambs are predicted to have poor survival), or poor ewe health [2,6,22,23,25].

Hogget breeding can be utilized in pastoral sheep production systems to increase the number of lambs produced each year, while concurrently increasing lifetime productivity [26,27,28]. However, there is a common concern among commercial farmers that hogget breeding may reduce ewe longevity, thereby increasing ewe wastage [29]. Despite this, there are only few studies that have evaluated this [2,26,29] with further investigation needed.

Body condition scoring of sheep is a quick, inexpensive and easily learned tool that was developed in the 1960s [30]. Body condition score (BCS) is used to assess the amount of subcutaneous fat and soft tissue by palpation of the lumbar region of the backbone spinous and transverse processes [30]. The relationship between BCS and various reproductive traits has been well documented, with the optimum BCS for a ewe from a production perspective suggested to be in the range of 2.5–3.5 at breeding (see review [31]). In addition, poor ewe BCS, particularly ewes with a BCS < 2.5, appears to be associated with increased ewe wastage, both in New Zealand and internationally [6,12,18]. For commercial farmers, it would be optimal if they were able to identify ewes that are likely to be poor performing or at greater risk of wastage, prior to breeding. If ewes with poor BCS were at greater risk of wastage, this would allow management interventions to be put in place to both improve performance and reduce risk of wastage.

The present study had three aims: firstly, to describe the extent, timing and general cause (premature culling or mortality) of ewe wastage in commercial New Zealand ewe flocks. Secondly, to investigate the association between reproductive outcomes as a hogget and risk of wastage. It was hypothesized that ewes that were bred as a hogget would have a greater risk of wastage. The final aim was to determine whether pre-mating body condition score (BCS) at each age could be used as a predictor of ewe wastage in that year, as it was hypothesized that ewes with poor BCS would have a greater risk of wastage.

## 2. Materials and Methods

### 2.1. Farms and Animals

The present study utilized data collected from 13,142 ewes during the period 2011–2017, as the ewes aged from replacement hoggets (7–8 months old) to 6-year-old ewes (Farms A and B) or to 3-year-old ewes (Farm C). All ewes that participated in this study were managed as part of three larger commercial flocks on New Zealand pastoral based sheep and beef farms (Farms A–C). Throughout this study, the flock managers did not change their normal management of their respective flocks, and continued to manage the study cohorts’ as part of the larger commercial flock.

Enrolment occurred when ewes were approximately 7–8 months of age, when each enrolled hogget was tagged with an electronic identification tag (EID; Alflex, Palmerston North, New Zealand) at this time. For each of the farms, all replacement hoggets present within the flock, for that respective year, were enrolled.

All farms were located in the North Island of New Zealand, and were convenience sampled. The farms were selected as they had large flock sizes (and would therefore have a large number of replacement hoggets), body condition scoring (BCS) and pregnancy diagnosis were already practiced on-farm, EID technology was already used in the flocks, and flock owners were interested in participating in a longitudinal study where data collection and reporting would continue for up to six years. Farms A and B routinely culled ewes for age following 6-year-old weaning. However, Farm C made a management decision to sell all remaining ewes in Year 3 (due to farm re-structure), therefore any ewes remaining in the Farm C cohort were censored at this time.

Farm A was located in the Waikato region, with a flock of semi-stabilized composite ewes consisting of Coopworth and East Friesian genotypes. Two cohorts of hoggets from Farm A were enrolled: 2010-born (*n* = 3717) and 2011-born (*n* = 4609). Farm B was located in the Wairarapa region and included Romney ewes that were 2011-born (*n* = 3998). Farm C was also located in the Wairarapa region and included Romney ewes that were 2014-born (*n* = 818).

The study met the ethical requirements of the Massey University Animal Ethics committee.

### 2.2. Reproductive Management

On Farm A and Farm C, all hoggets were joined with rams regardless of liveweight (7–8 months old at breeding). On Farm B, only selected hoggets (approximately 38 kg and above) were joined. In subsequent years, all ewes present on each farm were presented for breeding.

Each year, during mid-pregnancy, pregnancy diagnosis (PD) was undertaken and ewes were identified as either non-pregnant (dry, no fetus), single (one fetus) or multiple bearing (two or more fetuses). Following PD, dry, single and multiple bearing ewes were managed separately, to allow for their different feed requirements during pregnancy and lactation. Prior to lambing each year, ewes were set-stocked. Set-stocking involved allocating ewes into paddocks at a rate of approximately 6–12 ewes per hectare. During the lambing period, ewes from Farm A were observed every 2–3 days, while on Farm B and C they were observed daily by the farm staff. If seen, any obvious problems such as dystocia, vaginal prolapse or cast ewes were resolved. However, their incidence was not recorded, and no attempt was made to revive weak lambs or to mother-on or artificially rear orphaned lambs, as per normal practice on these farms.

Three to six weeks after parturition, the ewes and lambs were gathered into handling facilities for tail removal (tailing). At this time, the flock manager palpated the udder of each ewe and an assessment was made as to whether she was actively lactating (wet) or not (dry). Lambs were separated from the ewes (weaning) when the lambs were approximately 14–16 weeks old.

### 2.3. Grazing Management

As sheep in commercial flocks, the study ewes were grazed under typical New Zealand extensive pastoral conditions, on permanent pasture consisting of predominantly ryegrass (*Lolium perenne*) and white clover (*Trifolium repens*). Grazing decisions were made solely by the farmers to mimic commercial conditions, and no pasture measurements were taken. Ewes were rotationally grazed throughout the year, with the exception that they were set-stocked each year prior to lambing. Each year, on Farm B and Farm C set stocking occurred one to four weeks prior to the planned start of lambing. However, on Farm A, set stocking time varied between years; ewes were set stocked one to four weeks prior to the planned start of lambing in 2011, 2012 and 2015. However, in 2013, 2014, 2016 and 2017, ewes were set stocked immediately following PD.

### 2.4. Data Collection

During each study year, the researchers visited at four key management times: prior to breeding (pre-mating (PM)), at pregnancy diagnosis (PD), at set-stocking (where this occurred within four weeks of lambing; pre-lambing) and at weaning (W). A summary of the timing of each of the data collection visits, with reference to calendar date, management time, and time since enrolment (where Day 0 = day of enrolment), is presented in Appendix A. To enable standardization and subsequent comparison between cohorts, a mean ‘days since enrolment’ was assigned to each data collection visit, e.g., hogget PD was day 139 (Appendix A). This was calculated using the mean of Farm A 2010- and 2011-born and Farm B cohorts, but excluding Farm C as they were enrolled at a later management time (enrolment for Farm C occurred in the hogget PM-PD interval rather than immediately prior to hogget PM as in the other cohorts (Appendix A)).

#### 2.4.1. Body Condition Score (BCS)

At each management time, body condition score was assessed and recorded for all study ewes. Body condition scoring was undertaken by assessing the soft tissues over the lumbar region, using a 1–5 scale (where 1 = thin; 5 = obese), assessed to the nearest 0.5 of a BCS [30].

#### 2.4.2. Cull and Mortality Data

On each farm, ewes were culled at the flock managers’ discretion as per routine farm policy. At the time of culling, each ewe had her EID tag number, date and reason for culling recorded. On all farms, any ewe that was found dead had their EID tag collected and the interval in which they were found was recorded, with tags collected at the subsequent visit. However, data were not collected on every death due to the extensive nature of these farms and frequency of observation. Cause of death was not determined for any ewe.

### 2.5. Statistical Analyses

Data were stored in Microsoft Excel^®^, while all statistical analyses were conducted using either SAS (SAS Institute Inc., Cary, NC, USA, Version 9.4) or R (R, Version 3.6.0 for Windows, 64-bit).

#### 2.5.1. Lifetime Wastage and Related Descriptive Statistics

As this was a lifetime study, each ewe was assigned an outcome and corresponding ‘exit age’. By the end of this study, all ewes that had exited their respective flocks, were classified as either 1 = prematurely culled, or 2 = dead/missing, or if still in the flock, as 3 = censored, and either the exact date or interval in which they exited the flock was recorded. Ewe wastage is defined as the combination of classifications 1 and 2, i.e., premature culling and dead/missing, with dead/missing considered a proxy for on-farm mortality.

The assigned ‘exit age’ reflects the time each ewe remained in the flock following enrolment (Day 0). If a ewe was prematurely culled, then the date of culling was known, allowing assignment of an ‘exit age’ for that ewe. To allow for comparison between cohorts, this date was adjusted to correspond to the mean ‘days since enrolment’ used across all cohorts (Appendix A). However, if a ewe was classified as dead/missing, the exact date of exit was unknown. Therefore, if a ewe was absent from the last visit, and was not recorded as present at any of the subsequent visits or the tag was retrieved from a dead ewe, then it was classified as dead/missing in the interval between the last recorded date (i.e., the visit the ewe was recorded as present) and the visit immediately subsequent. This enabled the assignment of a ‘minimum exit age’ (last recorded date) and a ‘maximum exit age’ (visit immediately subsequent) for each of these ewes; thus, providing the interval they exited the flock. To allow for comparison between cohorts, the interval used the mean ‘days since enrolment’ (Appendix A).

As all farms culled ewes for age after the Year 6 weaning visit, any ewes present in the flock at this time were right-censored to reflect culling for age, rather than wastage. All ewes remaining in the Farm C cohort at Year 3 PD were sold to another farmer at that time point, and were therefore right-censored at that time to reflect this.

Set-stocking data were not collected for Farm A cohorts in some years (Appendix A) so to allow for this inconsistency the intervals used in reporting and analyses of wastage data were pre-mating to pregnancy diagnosis (PM-PD), pregnancy diagnosis to weaning (PD-W) and weaning to pre-mating (W-PM), as these were comparable across all study cohorts.

#### 2.5.2. Competing Risks Models

To account for competing risks of culling or dead/missing and interval-censored data, the survival analyses used semi-parametric competing risks regression under interval censoring, using the R package ‘intccr’ and the related methodology [32]. In the present analyses, the two competing events (outcomes) were wastage due to premature culling (c = 1), and wastage due to dead/missing (c = 2), with remaining ewes censored (c = 0).

Firstly, for each of the models (outlined below), a 10% random sample of the relevant dataset was selected, and four preliminary models were applied to the data. Each of these preliminary models returned only the estimated regression coefficients without calculating the bootstrap variance-covariance matrix for the estimated regression coefficients which was computationally intensive. The alternative preliminary models were subdistribution hazards models for both outcomes (c = 1, c = 2), proportional odds for both outcomes (c = 1, c = 2), subdistribution hazards for the first outcome (c = 1) and proportional odds for the second outcome (c = 2), and proportional odds for the first outcome (c = 1) and subdistribution hazards for the second outcome (c = 2). The model with the best fit was selected based on each model’s log likelihood. The final models used the entire dataset and 50 bootstrap samples to compute the variance-covariance matrix and obtain standard errors together with odds ratios and corresponding *p*-values. In addition, for the models examining the associations between pre-mating BCS and risk of ewe wastage, the mean cumulative incidence for each outcome (premature culling and dead/missing) were predicted for ewes from each cohort (Farm A 2010- and 2011-born, Farm B) on the basis of BCS (BCS 2.0 vs. BCS 3.5). These BCS (2.0 vs. 3.5) were chosen as they represent ewes that are considered to be poor BCS (BCS 2.0) and optimal BCS (BCS 3.5) for breeding.

##### Model for Association between Hogget Reproductive Outcomes and Risk of Subsequent Wastage

This model included only ewes from the Farm B cohort, as they were the only cohort that had both hoggets that were presented for breeding and hoggets that were not presented for breeding, and were the only cohort that did not cull any ewes based on reproductive outcomes as a hogget (i.e., all dry at PD and dry at tailing hoggets were retained in the flock). For a ewe to be included in the model, their reproductive outcome as a hogget had to be known and they had to be present in the flock at Year 2 (two tooth) pre-mating (the start point of the model). Ewes were classified into four categories based on hogget reproductive outcome: 1 = not presented for hogget breeding, 2 = presented for hogget breeding and dry at PD, 3 = pregnant as hogget but did not rear a lamb (dry at tailing), 4 = reared a lamb as a hogget. For this model, the predictor variable was hogget reproductive outcome. The final chosen model was proportional odds for both outcomes.

##### Model for Body Condition Score (BCS) as a Predictor of Wastage in That Production Year

For a ewe to be included in the model, they had to be present in the study cohort at the start of that year (pre-mating) and have a pre-mating BCS recorded. Each production year (pre-mating to following pre-mating) was analysed separately, with a production year defined as pre-mating to the subsequent pre-mating (i.e., pre-mating Year 1 to pre-mating Year 2). For each model, the predictor variable was pre-mating BCS, while each model also included additional covariates of cohort (Farm A 2010-born and 2011-born, Farm B, and Farm C). The final chosen model for each year was proportional odds for both outcomes.

## 3. Results

### 3.1. Lifetime Wastage and Descriptive Statistics

Of the 13,142 ewes enrolled in this study, 50.4% (*n* = 6629) exited their respective flocks due to premature culling, 40.0% (*n* = 5253) due to on-farm dead/missing, while only 5.1% (*n* = 676) were culled due to age (i.e., made it to the end of Year 6) and 4.4% (*n* = 584) were right censored (i.e., they were lost to the study), giving a total of 90.4% (*n* = 11,882) that exited due to wastage (Table 1).

**Figure 1:**
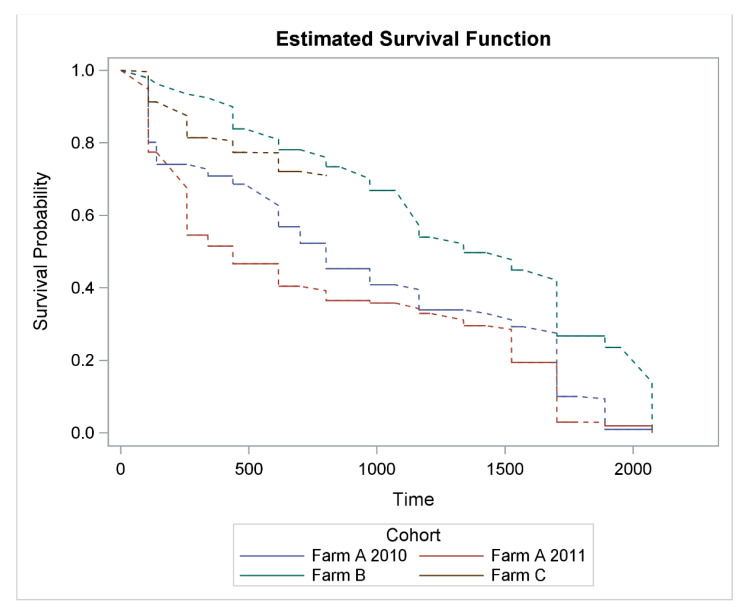
Interval-censored lifetime ewe wastage (combination of premature culling and on-farm mortality) from enrolment (time = Day 0) to Year 6 weaning (time = Day 2074), stratified by cohort (Farm A 2010-born and 2011-born, Farm B and Farm C). The dotted lines denote the uncertainty of when the ewes exited their respective flocks due to the interval-censored nature of the data.

Ewe wastage from enrolment to Year 6 weaning, where remaining ewes were culled for age, is shown in Figure 1. Actual recorded numbers of ewes that exited each cohort during each interval and production year are shown in Appendix B, which displays wastage of ewes over the whole study period. For both Farm A cohorts, wastage was greatest when ewes were younger and older, while for the Farm B cohort, the general trend was for wastage to increase as ewes aged. For the Farm C cohort, wastage was greater as hoggets, compared to two tooths. However, wastage could not be evaluated beyond this as all remaining ewes were censored in Year 3.

#### 3.1.1. Lifetime Wastage due to Premature Culling

An overall summary of recorded reasons for premature culling can be seen in Table 2, with 48.8% (*n* = 3231) of the prematurely culled ewes culled due to poor reproductive performance (non-pregnant, dry at PD; or failed to rear a lamb, dry at tailing). Of those 3231 ewes culled for poor reproductive performance, 32.5% (*n* = 1051) were culled for failure to rear a lamb, while the remainder were culled as they were non-pregnant (Table 2).

#### 3.1.2. Lifetime Wastage due to Dead/Missing

Ewe wastage due to dead/missing was greatest as hoggets for both Farm A cohorts, while for the Farm B cohort it was consistent across years, with the exception of Year 4 where it increased (Table 3). For the Farm C cohort, wastage due to dead/missing was only evaluated for hoggets (Year 1) and two tooths (Year 2) and four tooths until pregnancy diagnosis (Year 3 PD) (Table 3).

### 3.2. Ewe Wastage on an Annual Basis

Annual wastage incidence ranged from 7.6 to 90.0% in Years 1–5, and 35.8–91.2% in Year 6, with annual wastage greatest when ewes were older for the three cohorts that had ewes that remained (Figure 1; Table 4). This increased wastage in Year 5 was driven by premature culling—60.9%, 86.0% and 30.9% in the Farm A 2010, Farm A 2011 and Farm B cohorts, respectively (Appendix B). While the increased wastage in Year 6 was driven by premature culling for the Farm A 2010 cohort and mortality for the Farm A 2011 and Farm B cohorts (Appendix B). Actual recorded numbers of ewes that exited each cohort during each interval and production year are shown in Appendix B, which also displays ewe wastage on an annual basis (i.e., wastage of ewes that remained in the flock at the start of each interval and production year).

As hoggets, wastage for each cohort ranged from 7.6 to 45.4% of hoggets enrolled (Table 4; Appendix B). The Farm B cohort had the lowest wastage as hoggets (7.6%), but did not cull any hoggets for poor reproductive performance. In contrast, Farm A 2010-born and Farm C, respectively, culled 13.5% and 8.1% of enrolled hoggets for being non-pregnant, while Farm A 2011-born culled 16.7% of enrolled hoggets as they were non-pregnant and 11.0% as they failed to rear a lamb.

#### Annual Wastage due to Dead/Missing

Annual dead/missing incidence (a proxy for mortality rates) ranged from 3.5 to 20.8% in Years 1–5, with variation between cohorts and years (Table 5). For example, the Farm A 2010 cohort had annual mortality incidence of 12.8–13.9% from Years 1 to 4, with mortality decreasing to 8.7% in Year 5. In contrast, mortality incidence for the Farm B cohort were greatest in the older ewes. In Year 6, mortality incidence to weaning were 7.0% for the Farm A 2010 cohort and 24.8% and 40.2% for the Farm A 2011 and Farm B cohorts, respectively. In general, mortality incidences were greatest during the pregnancy diagnosis to weaning (PD-W) interval (Appendix B), with actual incidences reported in Table 6.

### 3.3. Association between Hogget Reproductive Outcome and Subsequent Risk of Wastage to Year 6 Weaning on Farm B

Ewes that were presented for breeding as a hogget but were non-pregnant as a hogget had 28.1% greater odds of wastage due to premature culling compared to ewes that were not bred as a hogget (OR = 1.281 (95%CI 1.166–1.397); *p* = 0.032).

There was no difference in risk of wastage due to premature culling of those that were bred as a hogget, pregnant at PD, but dry at tailing as a hogget (*p* = 0.471) or those that raised a lamb as a hogget (*p* = 0.818) compared to those that were not presented for breeding as a hogget. There was no association between reproductive outcomes as a hogget and subsequent wastage due to dead/missing (*p* > 0.2 for all groups).

### 3.4. Pre-Mating Body Condition Score (BCS) as a Predictor of Ewe Wastage in That Production Year (Pre-Mating to Subsequent Pre-Mating)

The number and percentage of ewes in each of the pre-mating BCS categories (1.0–5.0), at each year’s pre-mating visit, for each cohort, is available in Appendix C.

In addition to the OR reported below, the predicted mean cumulative incidence of wastage due to premature culling and wastage as a result of dead/missing were predicted for ewes on the basis of BCS (BCS 2.0 vs. BCS 3.5) (Table 7). Specifically, for Years 1–5, ewes that were BCS 2.0 had significantly greater mean cumulative incidence of wastage due to premature culling compared to ewes that were BCS 3.5. For Years 2, 3, 4 and 6, ewes that were BCS 2.0 had significantly greater mean cumulative wastage due to dead/missing compared to ewes that were BCS 3.5 (Table 7)).

#### 3.4.1. Year 1 (Hogget; 1-Year-Old at Lambing)

As a hogget, the odds of wastage due to premature culling were 31.2% lower (OR = 0.688 (95%CI 0.620–0.757); *p* < 0.0001) for each unit increase in pre-mating BCS. However, there was no association between pre-mating BCS and risk of wastage due to dead/missing (*p* = 0.431).

#### 3.4.2. Year 2 (Two Tooth; 2-Years-Old at Lambing)

As a two tooth, the odds of wastage due to premature culling were 57.0% lower (OR = 0.430 (95%CI 0.307–0.552); *p* < 0.0001) for each unit increase in pre-mating BCS, while the odds of wastage due to dead/missing were 24.4% lower (OR = 0.756 (95%CI 0.663–0.849); *p* = 0.003) for each unit increase in pre-mating BCS.

#### 3.4.3. Year 3 (Four Tooth; 3-Years-Old at Lambing)

As a four tooth, the odds of wastage due to premature culling were 86.2% lower (OR = 0.138 (95%CI 0.122–0.397); *p* < 0.0001) for each unit increase in pre-mating BCS, while the odds of wastage due to dead/missing were 34.8% lower (OR = 0.652 (95%CI 0.512–0.792); *p* = 0.002) for each unit increase in pre-mating BCS.

#### 3.4.4. Year 4 (Six Tooth; 4-Years-Old at Lambing)

As a six tooth, the odds of wastage due to premature culling were 43.4% lower (OR = 0.566 (95%CI 0.282–0.850); *p* = 0.045) for each unit increase in pre-mating BCS, while the odds of wastage due to dead/missing were 53.6% lower (OR = 0.464 (95%CI 0.313–0.616); *p* < 0.0001) for each unit increase in pre-mating BCS.

#### 3.4.5. Year 5 (Mixed-Age; 5-Years-Old at Lambing)

As a mixed-age ewe in Year 5, the odds of wastage due to premature culling were 36.7% lower (OR = 0.633 (95%CI 0.545–0.722); *p* < 0.0001) for each unit increase in pre-mating BCS. However, there was no association between pre-mating BCS and risk of wastage due to dead/missing (*p* = 0.336).

#### 3.4.6. Year 6 (Mixed-Age; 6-YEARS-OLD at lambing)

As a mixed-age ewe in Year 6, there was no association between pre-mating BCS and risk of wastage due to premature culling (*p* = 0.522). However, as a mixed-age ewe in Year 6, the odds of wastage due to dead/missing were 41.8% lower (OR = 0.582 (95%CI 0.383–0.781); *p* = 0.007) for each unit increase in pre-mating BCS.

## 4. Discussion

To the authors’ knowledge, this is the first study that reports both lifetime and annual wastage of ewes in a sample of commercial New Zealand flocks. Previous research has detailed either mortality or culling [15,16], but they appear not to have been considered together. Additionally, there are a limited number of international studies that have described ewe wastage [2,6,22,23]. Recent modelling demonstrated an increase in sheep flock productivity and subsequent profitability when wastage was reduced [1]. However, there is a need for sheep producers to have access to accurate estimates of wastage to provide a benchmark against which they can measure their own performance and gain greater clarity around the productive and economic impacts of reducing wastage in their flocks [1,2,22]. Before farmers can begin to reduce ewe wastage they need to understand the extent, timing, and cause of wastage in their commercial flocks, and risk factors associated with wastage, as explored in this study.

In the present study, we have identified ewe wastage as a potential issue for New Zealand commercial farmers, albeit on three farms, which when combined with previous results highlight the need for farmers to reduce wastage within their individual flocks if they wish to maximise overall productivity [1]. Both premature culling (particularly due to poor reproductive performance) and on-farm mortality (dead/missing) were identified as contributing to wastage, therefore both need to be considered when implementing strategies to reduce wastage.

Annual dead/missing incidence, considered a proxy for mortality rates, ranged from 3.5 to 20.8% in Years 1–5 (Table 5). This is comparable to previously reported annual on-farm mortality rates of 2.8–27.0% in New Zealand [15,16,18,19], 2.7–22.0% in Australian extensive flocks [11,12,20,33], and 3.0–10.0% for flocks based in the United Kingdom and Ireland [2,21,22,23]. However, in this study, mortality incidences in Year 6 were between 7.0 and 40.2% (Table 5). Higher mortality rates in older ewes (26%) have been reported in Australia [7]; however, the rates reported in six-year-old ewes in the present study are higher than those. These ewes are costly to the farmer as they have to be replaced, cull sale-value is not obtained, and there is an additional loss as there is the concurrent loss of her potential lamb(s) in that production year. In the present study, mortality incidences tended to be greatest during the pregnancy diagnosis to weaning interval. This agrees with previous reports of increased ewe mortality rates during the lambing period in both New Zealand and internationally [17,33,34,35]. It is also important to consider the welfare implications of having increased on-farm mortality rates [24], with mortality used in a number of welfare assessments [13].

As a hogget, annual wastage incidences were 7.6%, 18.3%, 27.3% and 45.4% for the Farm B, Farm C and Farm A 2010 and 2011 cohorts, respectively (Table 4). Rearing these replacement hoggets to a productive age would have incurred a number of costs [2,7], but given these ewes were lost to wastage prior to Year 2 breeding, it is unlikely they would have been in the flock long enough to recoup their rearing costs. In addition, hogget wastage due to dead/missing accounted for 26.8% (40/149; Farm C) to 100% (303/303; Farm B) of hogget wastage across cohorts (Appendix B), and premature culling was primarily due to poor reproductive performance (dry at pregnancy diagnosis or dry at tailing). Hence, other than the cull sale-value for those that were prematurely culled, the farmer received no productive or economic benefit from these wasted hoggets.

To reduce hogget wastage, the results of this study indicate a number of areas need to be considered. Firstly, in this study, hogget annual mortality incidences ranged from 4.9 to 17.7% (Table 5), while mortality in just the pregnancy diagnosis to weaning interval ranged from 4.4 to 13.4% (Table 6). These results indicate further investigation into the causes of and risk factors associated with hogget mortality in commercial flocks in required. Secondly, poor hogget reproductive performance results in both reduced productivity (less lamb produced per hogget presented for breeding) [36] and, as described in this study, increased premature culling (Farm A 2010, Farm A 2011 and Farm C). For the Farm A 2011 cohort in particular, culling decisions based on hogget reproductive performance resulted in a large number (1281/4609; 27.7%) of enrolled hoggets being prematurely culled in Year 1 (Appendix B). As described in this study, farmer culling decisions can have a significant impact on hogget wastage, therefore, if farmers elect to breed their hoggets and cull based on poor hogget reproductive performance they should address risk factors associated with poor reproductive performance.

Management practices required to maximise the likelihood of a hogget becoming pregnant are well documented for Romney-type sheep [29] and include a minimum liveweight of 40 kg (ideally greater than 65% of mature liveweight) and a recommended minimum BCS of 3.0 at breeding [27,29]. This contrasts with the management of hoggets in both Farm A and the Farm C cohorts, in which there was no minimum breeding weight applied, and with the Farm B cohort which used a minimum weight of approximately 38 kg. Once hoggets are pregnant, it is then important that losses to weaning be minimized, to both improve flock and individual ewe productivity and to reduce risk of premature culling due to being dry at tailing (failing to rear a lamb). However, evidence suggests losses of hogget lambs after pregnancy diagnosis continue to be an issue on New Zealand commercial farms [29,37,38]. A reduction in liveweight (after adjusting for conceptus weight, CALW) has been reported in hoggets prior to fetal loss, compared to hoggets that maintained their pregnancy [38]. Similarly, another study reported that hoggets with heavier CALW at pregnancy diagnosis and prior to lambing, hoggets with greater CALW gain between pregnancy diagnosis and prior to lambing, and hoggets with greater BCS at pregnancy diagnosis and prior to lambing, were less likely to be dry at tailing [39]. Combined, these results highlight the importance of monitoring hogget liveweight during pregnancy and accordingly adjusting feed intakes, to ensure hoggets continue to gain CALW.

It has been reported that commercial farmers are concerned that hogget breeding results in increased risk of subsequent wastage; however, there was limited published data evaluating this [2,26,29]. In the present study, the association between hogget reproductive outcomes and subsequent wastage were evaluated for the Farm B cohort, as they were the only cohort that had both hoggets that were presented for breeding and hoggets that were not presented for breeding, and which did not cull any hoggets for poor reproductive performance. Our data showed that pregnancy and/or lamb rearing as a hogget did not have any association with subsequent risk of wastage, due to either premature culling or mortality, when compared to those that were not presented for breeding as hoggets. This supports the conclusions that hogget breeding results in greater lifetime performance without negatively affecting ewe longevity [26,29]. However, it is important to note this may vary depending on overall hogget reproductive performance (i.e., no lifetime productive benefit if the hogget is dry at tailing) combined with an individual farm’s culling policies. For example, the Farm A 2011 cohort elected to cull hoggets that were dry at tailing, resulting in 16.4% (511/3116) of the cohort that remained at weaning being prematurely culled (Appendix B). Ewes from the Farm B cohort that were presented for breeding as hoggets but dry at pregnancy diagnosis had greater odds of premature culling compared to ewes that were not presented for breeding as hoggets. This suggests routine culling of hoggets that are dry at pregnancy diagnosis may be justified, as these ewes are more likely to be culled subsequently. However, further investigation of causes is required. It is also important to note, there was potential for bias in the Farm B cohort, as ewes that were presented for breeding were heavier than those that were not. In summary, further economic evaluation of culling on commercial farms based on hogget reproductive outcomes is required, as this represents a large area of wastage for some farms.

It would be optimal if commercial farmers could identify ewes that are at greater risk of wastage prior to breeding. This would allow either selective culling of these ewes earlier, enabling feed and resources to be directed to those ewes that are likely to be more productive [40], while obtaining cull sale-value, or would enable these ewes to be preferentially managed to reduce risk of wastage. The results of the present study support the hypothesis that ewes that had poorer pre-mating BCS would have a greater risk of wastage in that production year, with ewes with a BCS of 2.0 having a greater incidence of wastage compared to ewes that were BCS 3.5. There is a generally positive relationship between BCS and ewe reproductive traits and lamb survival [31]. In the present study, ewes with greater pre-mating BCS had lower odds of wastage due to premature culling. It is likely that these greater-BCS ewes had greater reproductive performance (i.e., were less likely to be dry at pregnancy diagnosis or tailing), and were therefore less likely to be prematurely culled. Greater BCS was associated with reduced risk of mortality in Years 2, 3, 4 and 6. This agrees with New Zealand results in which poor BCS was reported as a common cause of ewe mortality [18]. These results are also consistent with international data in which mortality rates were increased in poor-BCS ewes [6,12]. It is possible that for some of these poor-BCS ewes, the poor BCS is a proxy for other diseases [5,18,19]; therefore, further investigation into the underlying causes of poor BCS in commercial ewes is required. Combined, the above suggests farmers should focus on improving pre-mating BCS to reduce ewe wastage. From a production point of view, a pre-mating BCS of 2.5–3.5 has been recommended [31]; however, when considered alongside the results of the present study, a BCS of 3.5 would be preferable to reduce risk of wastage. To achieve this, farmers could assess the BCS of their ewes at weaning, enabling poor-BCS ewes to be preferentially managed to gain BCS before re-breeding. One unit of BCS is approximately 5–7 kg (depending on breed) [31], which will take time to gain, hence the recommendation to BCS at weaning. Since body condition scoring sheep is quick, inexpensive and easily learned [30,41], and doesn’t require any special equipment, it would be straightforward for farmers to implement on-farm.

The main limitations of this study arose from the extensive management of the commercial flocks and therefore limited frequency of interactions with individual ewes within the study cohorts which hindered acquiring complete data. There were only three or four on-farm data collection visits each year, resulting in collection of interval-censored data. However, these visits did occur at key management times for commercial sheep farms, and enabled a balance between collecting data on large commercial flocks and generating a robust dataset. Unfortunately, the extensive management of the flocks and frequency of observation combined with the paddock terrain meant it was not possible to collect data on every death, and the cause of death was not established for any ewe. Therefore, for analysis, missing ewes were classified in the same category as dead ewes (dead/missing), as it was presumed they were most likely dead. However, this was not accurately known. Although, given the study ewes were identified with EID tags and the study was conducted over a 6-year-period for each study cohort (with the exception of Farm C); it is likely that if these ewes were still present on the farm they would have had data collected at subsequent visits. The present study used data collected from only four cohorts, from three commercial farms. However, although there were differences in wastage described between the study cohorts, the general trends were comparable (for example increased ewe mortality from pregnancy diagnosis to weaning), and the relationships between wastage and BCS were consistent across cohorts. However, further studies involving more flocks and farms are required to increase the representation of flocks from the national population, to determine cause of on-farm mortalities and to investigate culling policies and their impacts on flock productivity and profitability, in order to provide more robust support for recommendations.

## 5. Conclusions

The present study outlined the incidence of wastage for four cohorts of ewe from three commercial farms as they aged from replacement hoggets to six-year-old ewes. Annual mortality incidence within a year group ranged from 3.5 to 40.2%, highlighting the need for further investigation into causes of, and risk factors associated with, ewe mortality in New Zealand extensive flocks. As a hogget, wastage incidence ranged from 7.6 to 45.4%, highlighting an area to target to reduce overall flock wastage. To reduce hogget wastage farmers should reduce on-farm hogget mortality, implement management practices to improve hogget reproductive performance, and evaluate (and adjust) their hogget culling policies. Body condition score (BCS) can be used as a predictor of wastage, with ewes with greater pre-mating BCS having lower odds of wastage due to both premature culling and mortality. Therefore, farmers should focus on improving pre-mating BCS, ideally achieving a BCS of 3.5/5.0, by assessing ewe BCS at weaning, allowing poorer-BCS ewes to be drafted off and managed to gain BCS before re-breeding.

## Figures and Tables

**Table 1:** Number and percentage (%) of ewes that were enrolled in this study and subsequently classified as dead/missing, prematurely culled or culled for age, following exit of all ewes from each cohort, for each enrolled cohort, and as an overall across cohorts. *n* represents total enrolled ewes for each cohort, and overall across cohorts.

	Farm A 2010	Farm A 2011	Farm B	Farm C ^1^	Overall
Total enrolled (*n*)	3717	4609	3998	818	13,142
Dead/Missing	1494	1515	2172	72	5253
% of *n*	40.2%	32.9%	54.3%	8.8%	40.0%
Prematurely culled	2190	3006	1271	162	6629
% of *n*	58.9%	65.2%	31.8%	19.8%	50.4%
Culled for age	33	88	555		676
% of *n*	0.9%	1.9%	13.9%		5.1%

^1^ Note, for the Farm C cohort, any ewes remaining at Year 3 PD (*n* = 584) were sold and were therefore right censored at this time.

**Table 2:** Lifetime wastage due to premature culling: of the ewes that were prematurely culled from their respective cohorts, for each cohort the number (and % of those that were prematurely culled within that cohort) that were recorded as culled for each reason, and the total across all study cohorts.

	Dry at PD	Dry at Tailing	Other ^1^	Unknown	Total
Farm A 2010	671 (30.6%)	210 (9.6%)	995 (45.4%)	314 (14.3%)	2190
Farm A 2011	918 (30.5%)	661 (22.0%)	1369 (45.4%)	58 (1.9%)	3006
Farm B	500 (39.3%)	151 (11.9%)	567 (44.6%)	53 (4.2%)	1271
Farm C ^2^	91 (56.2%)	29 (17.9%)	42 (25.9%)	0 (0.0%)	162
Total	2180 (32.9%)	1051 (15.9%)	2973 (44.8%)	425 (6.4%)	6629

^1^ Other includes ewes that were recorded as having been prematurely culled for poor teeth, poor feet, poor body condition score (BCS) or poor udder health. ^2^ Note, Farm C ewes were only included in the study to Year 3 pregnancy diagnosis.

**Table 3:** Number (and %) of total enrolled ewes that exited due to dead/missing during each year of the study, for each study cohort, and as an overall.

	Farm A 2010	Farm A 2011	Farm B	Farm C ^2^	Overall
Hogget, Y1 ^1^	513 (13.8%)	813 (17.7%)	303 (7.6%)	40 (4.9%)	1669 (12.7%)
Two tooth, Y2	345 (9.3%)	249 (5.4%)	314 (8.0%)	23 (2.8%)	931 (7.1%)
Four tooth, Y3	294 (7.9%)	149 (3.2%)	265 (6.7%)	9 (1.1%)	717 (5.5%)
Six tooth, Y4	210 (5.6%)	216 (4.7%)	555 (13.9%)		981 (7.5%)
Mixed age Y5	106 (2.9%)	54 (1.2%)	305 (7.7%)		465 (3.5%)
Mixed age Y6	26 (0.7%)	34 (0.7%)	430 (10.8%)		490 (3.7%)
Overall	1494 (40.2%)	1515 (32.9%)	2172 (54.3%)	72 (8.8%)	5253 (40.0%)

^1^ Y = year of study, for example Y1 = Year 1. ^2^ Note, Farm C ewes were only included in the study to Year 3 pregnancy diagnosis.

**Table 4:** Annual wastage: of the ewes that remained in each cohort at the start of each production year (pre-mating), the number (and percentage) of ewes that exited in that production year (Years 1–6) due to wastage (combination of premature culling and dead/missing), for each cohort.

	Farm A 2010	Farm A 2011	Farm B	Farm C ^2^
Hogget, Y1 ^1^	1014 (27.3%)	2094 (45.4%)	303 (7.6%)	149 (18.3%)
Two tooth, Y2	590 (21.9%)	651 (25.9%)	571 (15.5%)	76 (11.5%)
Four tooth, Y3	595 (28.1%)	213 (11.4%)	450 (14.4%)	
Six tooth, Y4	293 (19.3%)	288 (17.5%)	685 (25.7%)	
Mixed age Y5	852 (69.6%)	1226 (90.0%)	920 (46.2%)	
Mixed age Y6	340 (91.2%)	49 (35.8%)	514 (48.1%)	

^1^ Y = year of study, for example Y1 = Year 1. ^2^ Note, Farm C ewes were only included in the study to Year 3 pregnancy diagnosis.

**Table 5:** Annual dead/missing incidence (a proxy for annual mortality): of the ewes that remained in the cohort at the start of each production year (pre-mating), the number (and %) of ewes that exited due to dead/missing, for each study cohort.

	Farm A 2010	Farm A 2011	Farm B	Farm C ^2^
Hogget, Y1 ^1^	513 (13.8%)	813 (17.7%)	303 (7.6%)	40 (4.9%)
Two tooth, Y2	345 (12.8%)	249 (9.9%)	314 (8.5%)	23 (3.5%)
Four tooth, Y3	294 (13.9%)	149 (8.0%)	265 (8.5%)	
Six tooth, Y4	210 (13.8%)	216 (13.1%)	555 (20.8%)	
Mixed age Y5	106 (8.7%)	54 (4.0%)	305 (15.3%)	
Mixed age Y6	26 (7.0%)	34 (24.8%)	430 (40.2%)	

^1^ Y = year of study, for example Y1 = Year 1. ^2^ Note, Farm C ewes were only included in the study to Year 3 pregnancy diagnosis.

**Table 6:** Dead/missing from pregnancy diagnosis to weaning: of the ewes that the flock manager intended to retain until weaning (i.e., retain for lambing) ^1^, the number (and %) of ewes that exited due to dead/missing during the period from pregnancy diagnosis to weaning (PD-W), for each study cohort.

	Farm A 2010	Farm A 2011	Farm B	Farm C ^3^
Hogget, Y1 ^2^	383 (12.2%)	486 (13.4%)	178 (4.5%)	33 (4.4%)
Two tooth, Y2	231 (9.0%)	171 (7.4%)	157 (4.7%)	3 (0.5%)
Four tooth, Y3	199 (10.6%)	93 (5.2%)	166 (5.6%)	
Six tooth, Y4	131 (9.4%)	100 (6.5%)	111 (5.1%)	
Mixed age Y5	80 (7.3%)	7 (0.8%)	153 (8.3%)	
Mixed age Y6	2 (5.7%)	34 (27.9%)	425 (43.4%)	

^1^ Ewes that were culled before planned start of lambing (e.g., dry ewes) were excluded from the denominator, to better reflect the true mortality rate from pregnancy diagnosis to weaning. ^2^ Y = year of study, for example Y1 = Year 1. ^3^ Note, Farm C ewes were only included in the study to Year 3 pregnancy diagnosis.

**Table 7:** For each study year (Years 1–6), the predicted mean cumulative incidence from final multivariable models of wastage due to premature culling and wastage as a result of dead/missing for ewes from three cohorts (Farm A 2010, Farm A 2011, Farm B) on the basis of body condition score (BCS) (BCS 2.0 vs. BCS 3.5).

	Cohort	Premature Culling				Dead/Missing			
		BCS 2.0 ^1^	BCS3.5 ^2^	Diff ^3^	*p*-Value	BCS 2.0 ^4^	BCS 3.5 ^5^	Diff ^6^	*p*-Value
Year 1	A 2010	19.4%	12.1%	7.3%	<0.0001	13.0%	11.9%	1.1%	0.431
	A 2011	33.7%	22.5%	11.2%		19.0%	17.5%	1.5%	
	B	4.6%	2.7%	1.9%		10.3%	9.4%	0.9%	
Year 2	A 2010	16.7%	5.4%	11.3%	<0.0001	14.9%	10.3%	4.6%	0.003
	A 2011	25.4%	8.7%	16.7%		11.1%	7.6%	3.5%	
	B	12.5%	3.9%	8.6%		9.6%	6.5%	3.1%	
Year 3	A 2010	33.8%	2.5%	31.3%	<0.0001	16.4%	9.4%	7.0%	0.002
	A 2011	13.5%	0.7%	12.8%		10.4%	5.8%	4.6%	
	B	21.1%	1.4%	19.7%		10.1%	5.6%	4.5%	
Year 4	A 2010	8.2%	3.7%	4.5%	0.045	20.6%	7.6%	13.0%	<0.0001
	A 2011	7.7%	3.4%	4.3%		18.8%	6.8%	12.0%	
	B	8.8%	3.9%	4.9%		17.8%	6.4%	11.4%	
Year 5	A 2010	69.5%	53.5%	16.0%	<0.0001	8.3%	7.1%	1.2%	0.336
	A 2011	90.0%	81.9%	8.1%		3.7%	3.1%	0.6%	
	B	39.3%	24.6%	14.7%		16.5%	14.2%	2.3%	
Year 6	A 2010	81.6%	85.2%	−3.6%	0.522	1.1%	0.5%	0.6%	0.007
	A 2011	8.0%	10.1%	−2.1%		4.2%	1.9%	2.3%	
	B	6.7%	8.5%	−1.8%		11.1%	5.3%	5.8%	

^1^ The mean cumulative incidence of wastage due to premature culling for ewes from each cohort that had a pre-mating BCS of 2.0. ^2^ The mean cumulative incidence of wastage due to premature culling for ewes from each cohort that had a pre-mating BCS of 3.5. ^3^ The mean difference between BCS 2.0 estimate and BCS 3.5 estimate for wastage due to premature culling. ^4^ The mean cumulative incidence of wastage due to dead/missing for ewes from each cohort that had a pre-mating BCS of 2.0. ^5^ The mean cumulative incidence of wastage due to dead/missing for ewes from each cohort that had a pre-mating BCS of 3.5. ^6^ The mean difference between BCS 2.0 estimate and BCS 3.5 estimate for wastage due to dead/missing.

## Appendix A

**Table A1:** Summary of timing of each of the data collection visits, for each cohort of ewe (Farm A 2010-born, Farm A 2011-born, Farm B and Farm C) in a longevity study, with reference to: year-of-age (Years 1–6), management visit within each year (pre-mating, pregnancy diagnosis (PD), pre-lambing and weaning), calendar date, and time in days since enrolment (in brackets).

		Farm A 2010	Farm A 2011	Farm B	Farm C ^1^	Mean ^2^
Year 1	Pre-mating	11.4.11 (0)	11.4.12 (0)	1.4.12 (0)		0
	PD	14.7.11 (94)	23.7.12 (103)	2.8.12 (123)	27.7.15 (27)	107
	Pre-lambing	10.8.11 (121)	21.8.12 (132)	12.9.12 (164)	15.9.15 (77)	139
	Weaning	20.12.11 (253)	6.12.12 (239)	9.1.13 (283)	15.1.16 (199)	258
Year 2	Pre-mating	8.3.12 (332)	12.3.13 (335)	9.3.13 (352)	23.3.16 (267)	340
	PD	13.6.12 (429)	18.6.13 (433)	25.6.13 (450)	30.6.16 (366)	437
	Pre-lambing	16.7.12 (462)	-	20.8.13 (506)	22.8.16 (419)	484
	Weaning	13.12.12 (612)	19.12.13 (617)	6.12.13 (614)	12.12.16 (531)	615
Year 3	Pre-mating	13.3.13 (701)	11.3.14 (699)	3.3.14 (701)	17.3.17 (626)	700
	PD	17.6.13 (798)	18.6.14 (798)	17.6.14 (807)	20.6.17 (721)	801
	Pre-lambing	-	-	4.8.14 (855)		855
	Weaning	18.12.13 (982)	15.12.14 (978)	28.11.14 (955)		972
Year 4	Pre-mating	11.3.14 (1065)	11.3.15 (1064)	23.3.15 (1086)		1072
	PD	18.6.14 (1164)	9.6.15 (1154)	18.6.15 (1173)		1164
	Pre-lambing	-	20.7.15 (1195)	5.8.15 (1221)		1208
	Weaning	15.12.14 (1344)	7.12.15 (1335)	26.11.15 (1334)		1338
Year 5	Pre-mating	11.3.15 (1430)	2.3.16 (1421)	25.2.16 (1425)		1426
	PD	9.6.15 (1520)	6.6.16 (1517)	16.6.16 (1537)		1525
	Pre-lambing	20.7.15 (1561)	-	1.8.16 (1583)		1572
	Weaning	7.12.15 (1701)	13.12.16 (1707)	25.11.16 (1699)		1702
Year 6	Pre-mating	2.3.16 (1787)	28.2.17 (1784)	15.2.17 (1781)		1784
	PD	6.6.16 (1883)	8.6.17 (1884)	16.6.17 (1902)		1890
	Pre-lambing	-	-	4.8.17 (1951)		1951
	Weaning	13.12.16 (2073)	23.12.17 (2081)	28.11.17 (2067)		2074

^1^ Enrolment for Farm C occurred between pre-mating and pregnancy diagnosis, unlike in the other cohorts where it occurred prior to mating. ^2^ Mean days since enrolment, based on data from both Farm A cohorts and the Farm B cohort. -Data not collected for this visit, as the ewes were set-stocked for lambing at pregnancy diagnosis.

## Appendix B

**Table A2:** Number of ewes that were recorded as prematurely culled (C) or dead/missing (D) and overall wastage (W), during each interval in each year of the study, for each study cohort (Farm A 2010-born, Farm A 2011-born, Farm B and Farm C). PM-PD is the pre-mating to pregnancy diagnosis interval, PD-W is the pregnancy diagnosis to weaning interval, W-PM is the weaning to pre-mating interval, and Total is the production year (pre-mating to the subsequent pre-mating). The percentage of total enrolled (% TE) and the percentage of the remaining cohort (% R) are also provided for each year; where the % TE represented the percentage of ewes that were recorded in each category of those enrolled in the study (enrolled), and % R represents the percentage of ewes that were recorded in each category of those present at the start of the year.

		Farm A 2010				Farm A 2011				Farm B				Farm C			
			C	D	W		C	D	W		C	D	W		C	D	W
YI	Enrolled	3717				4609				3998				818			
	PM-PD		0	79	79		0	237	237		0	82	82				
	PD-W		501	383	884		770	486	1256		0	178	178		66	33	99
	W-PM		0	51	51		511	90	601		0	43	43		43	7	50
	Total		501	513	1014		1281	813	2094		0	303	303		109	40	149
	% TE		13.5	13.8	27.3		27.7	17.7	45.4		0	7.6	7.6		13.4	4.9	18.3
	% R		13.5	13.8	27.3		27.7	17.7	45.4		0	7.6	7.6		13.4	4.9	18.3
Y2	Start	2703				2515				3695				669			
	PM-PD		2	67	69		96	43	139		0	98	98		0	7	7
	PD-W		70	231	301		56	171	227		204	157	361		24	3	27
	W-PM		173	47	220		250	35	285		53	59	112		29	13	42
	Total		245	345	590		402	249	651		257	314	571		53	23	76
	% TE		6.7	9.3	16.0		8.7	5.4	14.1		6.4	8.0	14.4		6.4	2.8	9.2
	% R		9.1	12.8	21.9		16.0	9.9	25.9		7.0	8.5	15.5		8.0	3.5	11.5
Y3	Start	2113				1864				3124				593			
	PM-PD		129	39	168		0	56	56		0	85	85		0	9	9
	PD-W		62	199	261		33	93	126		70	166	236				
	W-PM		110	56	166		31	0	31		115	14	129				
	Total		301	294	595		64	149	213		185	265	450				
	% TE		8.2	7.9	16.1		1.4	3.2	4.6		4.7	6.7	11.4				
	% R		14.2	13.9	28.1		3.4	8.0	11.4		5.9	8.5	14.4				
Y4	Start	1518				1651				2674							
	PM-PD		0	48	48		0	72	72		0	385	385				
	PD-W		78	131	209		43	100	143		94	111	205				
	W-PM		5	31	36		29	44	73		36	59	95				
	Total		83	210	293		72	216	288		130	555	685				
	% TE		2.2	5.6	7.8		1.6	4.7	6.3		3.3	13.9	17.2				
	% R		5.5	13.8	19.3		4.4	13.1	17.5		4.9	20.8	25.7				
Y5	Start	1225				1363				1989							
	PM-PD		40	26	66		1	47	48		0	78	78				
	PD-W		57	80	137		413	7	420		75	153	228				
	W-PM		649	0	649		758	0	758		540	74	614				
	Total		746	106	852		1172	54	1226		615	305	920				
	% TE		20.1	2.9	23.0		25.4	1.2	26.6		15.4	7.7	23.1				
	% R		60.9	8.7	69.6		86.0	4.0	90.0		30.9	15.3	46.2				
Y6	Start	373				137				1069							
	PM-PD		0	24	24		4	0	4		0	5	5				
	PD-W		314	2	316		11	34	45		84	425	509				
	Total		314	26	340		15	34	49		84	430	514				
	% TE		8.4	0.7	9.1		0.3	0.7	1.1		2.1	10.8	12.9				
	% R		84.2	7.0	91.2		10.9	24.8	35.8		7.9	40.2	48.1				
End ^1^	Age				33				88				555				

^1^ The ewes that remained at the end of the study (Year 6 weaning) were culled due to age; *n* = 33, *n* = 88 and *n* = 555 for Farm A 2010, Farm A 2011 and Farm B cohorts, respectively.

## Appendix C

**Table A3:** Of the ewes that had a pre-mating body condition score (BCS) recorded ^1^, the number (N) and percentage (%) of ewes in each of the BCS categories (1.0–5.0), for each enrolled cohort (Farm A 2010, Farm A 2011, Farm B and Farm C) for each study year.

		Farm A 2010		Farm A 2011		Farm B		Farm C	
		N	%	N	%	N	(%)	N	%
Year 1	1.0	0	(0.0%)	0	(0.0%)	0	(0.0%)		
	1.5	23	(0.7%)	0	(0.0%)	1	(0.03%)		
	2.0	463	(13.4%)	16	(0.4%)	50	(1.34%)		
	2.5	1288	(37.1%)	1502	(32.9%)	1533	(41.2%)		
	3.0	1101	(31.7%)	2264	(49.5%)	1739	(46.8%)		
	3.5	490	(14.1%)	715	(15.6%)	361	(9.7%)		
	4.0	88	(2.5%)	71	(1.6%)	32	(0.9%)		
	4.5	16	(0.5%)	4	(0.1%)	3	(0.1%)		
	5.0	0	(0.0%)	0	(0.0%)	0	(0.0%)		
	Total	3469		4572		3719			
Year 2	1.0	0	(0.0%)	0	(0.0%)	0	(0.0%)	0	(0.0%)
	1.5	2	(0.1%)	4	(0.2%)	0	(0.0%)	0	(0.0%)
	2.0	185	(7.0%)	90	(3.7%)	103	(2.8%)	35	(5.2%)
	2.5	902	(33.9%)	1210	(49.2%)	1566	(43.2%)	268	(39.9%)
	3.0	936	(35.2%)	1076	(43.8%)	1860	(51.3%)	296	(44.1%)
	3.5	435	(16.3%)	76	(3.1%)	100	(2.8%)	65	(9.7%)
	4.0	154	(5.8%)	3	(0.1%)	0	(0.0%)	5	(0.8%)
	4.5	43	(1.6%)	0	(0.0%)	0	(0.0%)	0	(0.0%)
	5.0	0	(0.0%)	0	(0.0%)	0	(0.0%)	0	(0.0%)
	Total	2662		2459		3629		669	
Year 3	1.0	0	(0.0%)	1	(0.1%)	0	(0.0%)	0	(0.0%)
	1.5	2	(0.1%)	48	(2.6%)	8	(0.3%)	0	(0.0%)
	2.0	218	(10.6%)	652	(35.5%)	87	(2.9%)	10	(1.7%)
	2.5	1136	(55.3%)	965	(52.5%)	1063	(35.1%)	151	(26.0%)
	3.0	634	(30.9%)	173	(9.4%)	1645	(54.3%)	343	(59.0%)
	3.5	60	(2.9%)	0	(0.0%)	217	(7.2%)	74	(12.7%)
	4.0	4	(0.2%)	0	(0.0%)	11	(0.4%)	3	(0.5%)
	4.5	0	(0.0%)	0	(0.0%)	1	(0.03%)	0	(0.0%)
	5.0	0	(0.0%)	0	(0.0%)	0	(0.0%)	0	(0.0%)
	Total	2054		1839		3032		581	
Year 4	1.0	1	(0.1%)	0	(0.0%)	0	(0.0%)		
	1.5	1	(0.1%)	0	(0.0%)	1	(0.04%)		
	2.0	59	(4.0%)	17	(1.1%)	30	(1.3%)		
	2.5	524	(35.5%)	202	(12.8%)	356	(15.5%)		
	3.0	665	(45.1%)	837	(53.0%)	1171	(50.8%)		
	3.5	216	(14.6%)	492	(31.1%)	716	(31.1%)		
	4.0	10	(0.7%)	32	(2.0%)	30	(1.3%)		
	4.5	0	(0.0%)	0	(0.0%)	0	(0.0%)		
	5.0	0	(0.0%)	0	(0.0%)	0	(0.0%)		
	Total	1476		1580		2304			
Year 5	1.0	1	(0.1%)	0	(0.0%)	0	(0.0%)		
	1.5	1	(0.1%)	3	(0.2%)	14	(0.7%)		
	2.0	28	(2.3%)	20	(1.5%)	251	(13.0%)		
	2.5	352	(29.2%)	210	(15.6%)	789	(40.8%)		
	3.0	600	(49.8%)	883	(65.6%)	747	(38.6%)		
	3.5	204	(16.9%)	219	(16.3%)	126	(6.5%)		
	4.0	20	(1.7%)	11	(0.8%)	8	(0.4%)		
	4.5	0	(0.0%)	0	(0.0%)	0	(0.0%)		
	5.0	0	(0.0%)	0	(0.0%)	0	(0.0%)		
	Total	1206		1346		1935			
Year 6	1.0	1	(0.3%)	1	(1.0%)	0	(0.0%)		
	1.5	7	(1.9%)	0	(0.0%)	0	(0.0%)		
	2.0	32	(8.8%)	0	(0.0%)	2	(0.2%)		
	2.5	141	(39.0%)	8	(7.8%)	69	(6.6%)		
	3.0	147	(40.6%)	72	(70.0%)	607	(57.8%)		
	3.5	30	(8.3%)	21	(20.4%)	312	(29.7%)		
	4.0	4	(1.1%)	1	(1.0%)	61	(5.8%)		
	4.5	0	(0.0%)	0	(0.0%)	0	(0.0%)		
	5.0	0	(0.0%)	0	(0.0%)	0	(0.0%)		
	Total	362		103		1051			

^1^ Note, not all ewes that were present at each time point had body condition score (BCS) data records available.
